# Type III Effector Diversification via Both Pathoadaptation and Horizontal Transfer in Response to a Coevolutionary Arms Race

**DOI:** 10.1371/journal.pgen.0020209

**Published:** 2006-12-22

**Authors:** Wenbo Ma, Frederick F. T Dong, John Stavrinides, David S Guttman

**Affiliations:** 1 Department of Cell and Systems Biology, University of Toronto, Toronto, Ontario, Canada, **2** Centre for the Analysis of Genome Evolution and Function, University of Toronto, Toronto, Ontario, Canada; Fred Hutchinson Cancer Research Center, United States of America

## Abstract

The concept of the coevolutionary arms race holds a central position in our understanding of pathogen–host interactions. Here we identify the molecular mechanisms and follow the stepwise progression of an arms race in a natural system. We show how the evolution and function of the HopZ family of type III secreted effector proteins carried by the plant pathogen Pseudomonas syringae are influenced by a coevolutionary arms race between pathogen and host. We surveyed 96 isolates of P. syringae and identified three homologs (HopZ1, HopZ2, and HopZ3) distributed among ∼45% of the strains. All alleles were sequenced and their expression was confirmed. Evolutionary analyses determined that the diverse HopZ1 homologs are ancestral to P. syringae, and have diverged via pathoadaptive mutational changes into three functional and two degenerate forms, while HopZ2 and HopZ3 have been brought into P. syringae via horizontal transfer from other ecologically similar bacteria. A PAML selection analysis revealed that the C terminus of HopZ1 is under strong positive selection. Despite the extensive genetic variation observed in this family, all three homologs have cysteine–protease activity, although their substrate specificity may vary. The introduction of the ancestral *hopZ1* allele into strains harboring alternate alleles results in a resistance protein-mediated defense response in their respective hosts, which is not observed with the endogenous allele. These data indicate that the P. syringae HopZ family has undergone allelic diversification via both pathoadaptive mutational changes and horizontal transfer in response to selection imposed by the host defense system. This genetic diversity permits the pathogen to avoid host defenses while still maintaining a virulence-associated protease, thereby allowing it to thrive on its current host, while simultaneously impacting its host range.

## Synopsis

Pathogens and their hosts impose reciprocal selective pressures on each other, such that the improvement of one selects for the improvement of the other. Pathogens that are able to evolve increasingly effective methods of attacking their hosts select for hosts that are able to mount increasingly effective defenses against pathogen attack. This coevolutionary interaction is commonly referred to as an arms race, or the Red Queen Principle, taken from Lewis Carroll's *Through the Looking Glass, and What Alice Found There,* in which Alice and the Red Queen had to run as fast as they could simply to stay in the same place. Many pathogenic bacteria rely on specialized virulence proteins, called type III secreted effectors (T3SEs), to cause disease. These proteins are injected into the cells of the host, and often act to disrupt the host defense response. This study shows how the HopZ family of T3SEs in the pathogen Pseudomonas syringae evolves in response to coevolutionary selective pressures imposed by its plant hosts. The authors identify the version of the *hopZ* gene that is most similar to the one carried by the ancestral strain, and then show how this version has been modified by mutation and selection in response to the host defense systems. They also identify genes related to *hopZ* from other species that were brought into P. syringae presumably in response to this same host-imposed selective pressure. Finally, the authors show how the genetic diversity in this gene family permits the pathogen to avoid host defenses while still maintaining an important virulence-associated function. This study provides a clearer picture of the molecular interactions that drive coevolutionary interactions, and insight into how ecological processes play out at the molecular and evolutionary scale.

## Introduction

The paradigm of the coevolutionary arms race has had a tremendous influence on our interpretation of pathogen–host interactions [1]. It has profoundly influenced our understanding of the mechanisms by which genetic variation is maintained in populations, and how variation in one species influences the evolutionary trajectory of interacting species [2,3]. Because of this central role, it is important to dissect the molecular basis and dependencies of this process to fully understand the evolutionary forces and molecular and ecological interactions that drive the stepwise escalatory adaptations that are the hallmark of coevolutionary arms races.

Bacterial virulence proteins secreted through the type III secretion system (T3SS) and the host defense proteins that respond to them are likely instigators and agents of arms races. The T3SS is a specialized injection apparatus used by many Gram-negative bacteria to directly deliver bacterial type III secreted effectors (T3SEs) into the cytosol of their eukaryotic hosts [4]. T3SEs play integral roles in bacterial virulence by promoting pathogen growth [5] and suppressing host defense pathways [6]. Due to their central role in pathogenesis and direct and intimate interactions with substrates inside the host cell, T3SEs impose strong selective pressures on the host [7], while being exposed to reciprocal selective pressures imposed by the host defense systems [8]. Plant hosts have responded to these bacterial challenges by evolving resistance (R) proteins that trigger a defense response upon recognition of specific T3SEs [9]. Pathogens may then respond by modifying or even losing the T3SE so that they are no longer detected [10]. In this manner, bacterial T3SEs and plant R proteins are engaged in a classic coevolutionary arms race [2,3,11].

One of the most diverse and widely distributed families of T3SEs is the Yersinia pestis YopJ family of cysteine proteases [12]. Members of this large family of T3SEs are found among both animal and plant pathogens, implying that they may target conserved eukaryotic substrates. YopJ homologs have been shown to suppress immune responses in both animal and plant hosts, either by mimicking the enzymatic activity of the small ubiquitin-related modifier protease inside eukaryotic cells, or by inhibiting MAPK and NFκB defense signaling pathways [13–15].

In this study we show how YopJ homologs in Pseudomonas syringae, which are referred to as the HopZ family in this species, mediate a classic arms race with their plant hosts. P. syringae is an agronomically important plant pathogenic γ-proteobacterium that causes disease in a wide variety of important crop species. While the host range of the P. syringae species is vast, individual isolates typically cause disease in a very restricted set of hosts.

Three YopJ homologs have been identified in P. syringae [16]: HopZ1 (formerly HopPsyH and HopPmaD), HopZ2 (formerly AvrPpiG), and HopZ3 (formerly HopPsyV). HopZ1 was originally identified in the ornamental pear pathogen, P. syringae pv. *syringae* A2 (PsyA2) [17] and the radish pathogen, P. syringae pv. *maculicola* ES4326 (PmaES4326) [18]. HopZ2 was originally identified in the pea pathogen, P. syringae pv. *pisi* 895A (Ppi895A). All known *hopZ2* alleles are located on plasmids and flanked by mobile elements [19]. HopZ3 was originally isolated from the bean pathogen, P. syringae pv. *syringae* B728a (PsyB728a) [20,21]. This T3SE is found in the highly variable exchange effector locus, which lies to one side of the *hrp/hrc* cluster that encodes the T3SS in P. syringae. HopZ3 was recently shown to suppress the defense signaling-associated cell death in Nicotiana benthamiana, yet it intriguingly acts as an avirulence factor both in this host and in snap bean [22]. Additionally, it facilitates bacterial growth, and thereby acts as a virulence factor in Arabidopsis thaliana [22]. All three of these T3SEs share the cysteine-protease catalytic core consisting of the histidine, glutamic acid, and cysteine residues required for proteolytic function [12].

Previous studies have demonstrated that the inactivation of T3SEs from P. syringae and other plant pathogens in agricultural or contrived conditions can profoundly influence pathogen–host interactions [11,23–26]. Most of this work has implicated horizontal gene transfer and the resulting acquisition and loss of T3SE as an important arms race strategy [27,28]. The alternative to modulating virulence via horizontal gene transfer is pathoadaptation, in which increased virulence is achieved through relatively minor mutations (e.g., point mutations) in preexisting genes [29–32]. While the evolutionary signature of positive selection has been identified in T3SEs [33], the diversification of T3SEs via pathoadaptive mutational changes has not yet been shown to influence pathogen–host interactions, except in fairly specialized cases when the variation has been introduced by strand-slippage repeat expansion [34,35] or the insertion of mobile elements [36–38]. The P. syringae T3SE *avrPphE* presents a strong case for the importance of mutation in T3SE-mediated pathogen–host interactions, although the support for pathoadaptation in this system is equivocal since the genes were overexpressed using strong constitutive promoters [39].

Here we show through the sampling and analysis of extant alleles from natural populations of P. syringae that the HopZ family of T3SEs was present in an early ancestor of the species, and that coevolutionary interactions with the host defense system have resulted in allelic diversification via both pathoadaptive mutation and horizontal gene transfer from ecologically similar bacteria. We demonstrate that this has ultimately resulted in a highly polymorphic T3SE family, with clear implications for P. syringae host specificity.

## Results/Discussion

### Evolutionary Relationships of P. syringae HopZ Homologs

**Figure 1:**
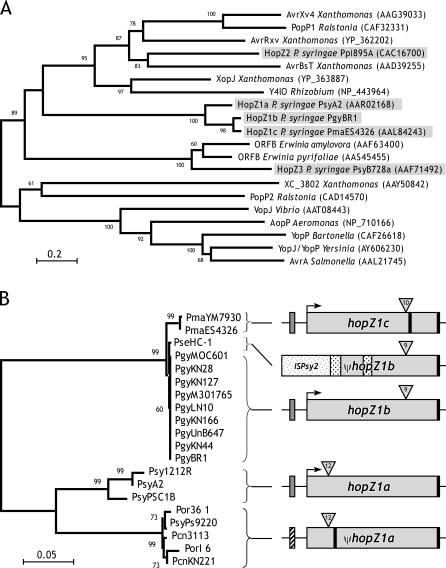
Phylogenetic Analyses (A) Neighbor-joining tree of YopJ family of T3SEs proteins. Bootstrap support is indicated above each node, with only values >60% being shown. T3SEs from P. syringae are highlighted. Accession numbers for each protein are presented in parentheses following the protein name and species. (B) Neighbor-joining gene genealogy of the *hopZ1* T3SEs alleles with bootstrap analysis as above and *hopZ2_Ppi895A_* used as an out-group. The genetic organization of the three functional allele classes (*hopZ1a*, *hopZ1b,* and *hopZ1c*) and two degenerate *hopZ1* alleles (ψ*hopZ1a* and ψ*hopZ1b*) are presented to the right of the gene genealogy. The large gray rectangle represents the region of shared similarity among the alleles, and corresponds to the coding sequence of *hopZ1a*. The dark vertical rectangle to the left of each gene represents the T3SS promoter element known as the *hrp* box. The solid black vertical line represents the stop codon for each allele. Arrows at the 5′ end of the coding sequences indicate which alleles are functional. Triangles above each coding sequence indicate insertions, with the insertion size indicated within. The ψ*hopZ1a* allele has a nonsense mutation at nucleotide 171, and a degenerate *hrp* box in which the conserved GGAACC sequence has mutated to CCAACC. It is not transcribed under T3SS induction conditions. The ψ*hopZ1b* allele is disrupted by an insertion sequence.

A phylogenetic analysis of the complete YopJ family places the P. syringae effectors in three distinct clades (Figure 1A). The HopZ1 alleles form a distinct clade restricted to P. syringae. HopZ2 clusters with T3SEs from the tomato and pepper pathogen, Xanthomonas campestris (AvrBsT, AvrRxv, and AvrXv4). HopZ3 is in a distinct clade with the virulence-associated ORFB locus from the Asian pear pathogen, *Erwinia pyrifoliae,* and the fire blight pathogen, Erwinia amylovora. These phylogenetic relationships suggest that *hopZ1* is native to P. syringae, while the clustering of *hopZ2* and *hopZ3* within clades dominated by *Xanthomonas* and *Erwinia* T3SEs, respectively, supports the argument that these T3SEs may be functionally related homologs that were horizontally acquired from ecologically related plant pathogens.

**Figure 2:**
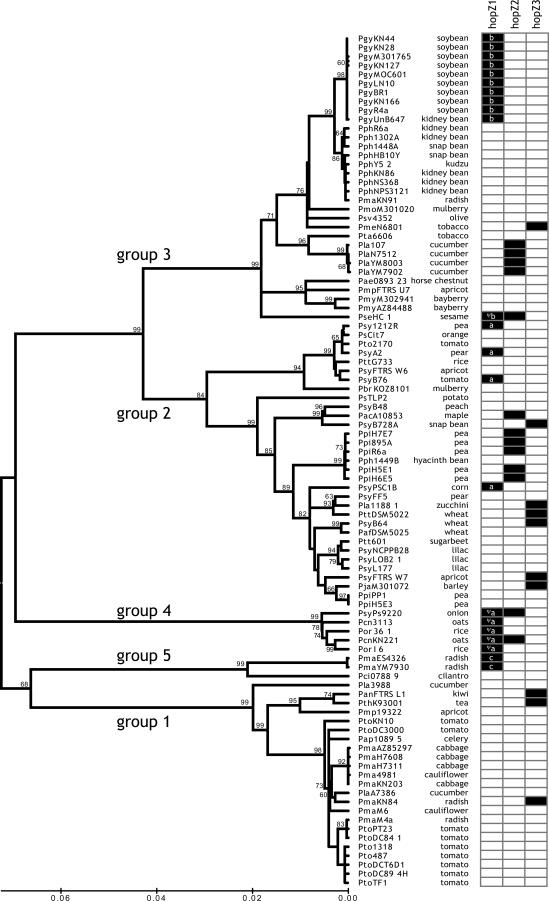
Distribution of HopZ T3SEs in the P. syringae Species Complex The distribution of HopZ T3SEs in relation to the core genome phylogeny of 96 natural P. syringae isolates. The phylogenetic tree (neighbor-joining, 1,000 bootstrap) was generated by MLST of four housekeeping genes. The host of isolation is presented to the right of each strain. See [40,65] and Table S1 for details. Black squares represent presence of the corresponding gene, whereas white ones represent absence. *hopZ1* alleles are annotated. The full sequence of *hopZ1a_PsyB76_* was not obtained although its expression was confirmed.

The distribution of the HopZ T3SEs was examined in our collection of P. syringae strains isolated worldwide from ∼40 plant hosts. All 96 strains were phylogenetically characterized via multilocus sequence typing (MLST) [40] of the core genome to determine their clonal evolutionary relationships. Both Southern hybridization using T3SE probes and PCR were used to determine the distribution of the HopZ family members. Forty-two isolates, distributed throughout all five P. syringae phylogroups [40], were positive for at least one effector (Figure 2). Promoter analysis, RT-PCR, and northern blot analysis (Figure S1) confirmed that a total of 39 isolates from 18 different hosts express *hopZ*, with 16 strains expressing *hopZ1*, 13 expressing *hopZ2*, and ten expressing *hopZ3*. No isolates expressed more than one HopZ T3SE, although three strains expressing *hopZ2* also carry degenerate forms of *hopZ1*. The frequency and broad distribution of the HopZ T3SEs suggest that these genes were present in an early ancestor of P. syringae.

### HopZ1a Is the Ancestral Allelic Form

A relative-rates test of all *hopZ* homologs (Figure S2) reveals that the three major forms are evolving in approximately a clock-like manner (unpublished data); however, *hopZ1* has significantly higher overall average pairwise nucleotide diversity (π) at 0.149 ± 0.034 (sd), versus 0.032 ± 0.006 and 0.096 ± 0.035 for *hopZ2* and *hopZ3,* respectively (*p* < 0.0001 for all unpaired *t*-test comparisons), as well as the highest synonymous pairwise nucleotide diversity (0.234, 0.087, and 0.193, for *hopZ1, hopZ2,* and *hopZ3,* respectively). The clock-like evolution of *hopZ1* suggests that its higher genetic diversity is due to a greater age in the population relative to *hopZ2* and *hopZ3*, rather than an accelerated pace of evolution. This supports the hypothesis that *hopZ1* is more ancestral than *hopZ2* and *hopZ3,* and that these latter two homologs were brought into P. syringae via horizontal transfer after the species began to diversify.

HopZ1 has three functional and two degenerate allelic forms (Figure 1B). Of the functional alleles, *hopZ1b* and *hopZ1c* differ from *hopZ1a* in that they have a 12-base-pair (bp) deletion encompassing amino acids 31–34, which removes a predicted alpha helix [41]. *hopZ1b* also has a 9-bp insertion near its 3′ end, while *hopZ1c* has a 10-bp insertion at the same site. The insertion in *hopZ1c* causes a frameshift that prematurely terminates translation (Figure S3). Although HopZ1c is approximately 25% shorter than HopZ1a, it still retains the protease catalytic triad and its enzymatic activity (see below). The two degenerate *hopZ1* alleles were found in six isolates. One of these alleles is due to a nonsense mutation at nucleotide 171, while the other is due the integration of an insertion sequence. The alleles are not expressed under either T3SS-inducing or rich media.

**Figure 3:**
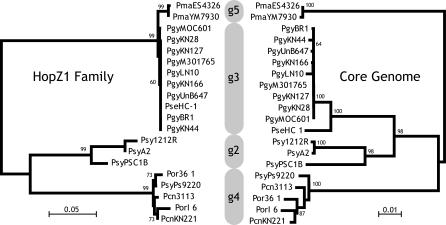
Congruence between the HopZ1 Gene Genealogy and the MLST Core Genome Phylogeny of P. syringae See Figures 2 and S1 for details on the phylogenetic methods used. Gray areas labeled g2–g5 in the center of the figure indicate the major phylogroups as determined by MLST. Bootstrap support values (>60%) are indicated above nodes.

The clustering of alleles in the *hopZ1* gene genealogy is consistent with the major clades (phylogroups) seen in the P. syringae MLST core-genome tree (Figure 3). The single large-scale incongruence between the *hopZ1* gene tree and the core genome phylogeny is the tight clustering between the *hopZ1b* alleles from phylogroup 3 strains and the *hopZ1c* alleles from phylogroup 5 strains. Specifically, it appears as if *hopZ1* alleles from phylogroup 5 strains transferred into strains of phylogroup 3. An identical incongruity between the phylogroup 3 and 5 strains was observed for one of the major operons that encodes the T3SS apparatus [42]. These results support a horizontal gene exchange event between phylogroups 3 and 5 that mobilized at least part of the genes encoding the T3SS and some of its effectors. There are also some minor points of incongruence within the major clades of the *hopZ1* gene genealogy with respect to the MLST core-genome tree, but none of these are strongly supported by bootstrap analysis in both trees. An SH phylogenetic congruence test [43] for the phylogroup 2, 4, and 5 strains (phylogroup 3 strains were omitted in this analysis for the reason discussed above) also finds that the MLST data is congruent with the HopZ1 tree (unpublished data).

The congruence between the MLST core-genome tree and the HopZ1 tree implies that the genes used in these analyses have a shared evolutionary history. As discussed above, *hopZ1* is restricted to P. syringae and has significantly greater diversity than *hopZ2* or *hopZ3.* Furthermore, *hopZ1a* falls in the center of a network phylogenetic analysis (Figure S4). These lines of evidence indicate that a *hopZ1a*-like allele was present in an early ancestor of P. syringae and that the other *hopZ1* alleles are mutational derivatives of *hopZ1a*.

### HopZ1 Alleles Are under Strong Positive Selection

**Figure 4:**
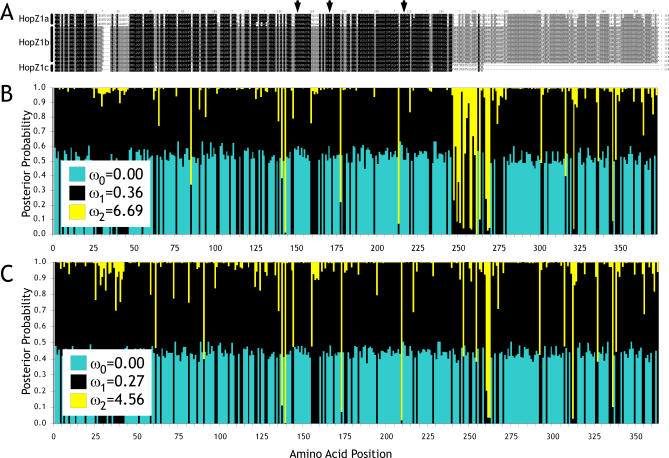
PAML Selection Analysis (A) Amino-acid alignment of the HopZ1 alleles. See Figure S3 for details. The three amino acids in the catalytic triad are indicated by arrows presented above the alignment. (B) Stacked histogram representing the posterior probabilities for the three site classes identified by codeml model M3 (discrete) along the HopZ1 sequence for all functional alleles. The dN/dS (ω) values for each site class are given below the figure. (C) Same as above but using only *hopZ1a* and *hopZ1b* to remove the signal from the frameshift mutation in *hopZ1c*.

If *hopZ1a* was carried by an early ancestor of *P. syringae,* it has been lost in many lineages, replaced by mutational derivatives in others, or even substituted for by homologs through horizontal transfer. This is most likely due to selection on HopZ1 for alternative alleles imposed by an arms race. We use the codeml module of PAML [44] to test for selection. PAML is a likelihood-based application for estimating synonymous and nonsynonymous substitution rates and testing various models of evolution in DNA and protein sequences. Codeml can be used to determine the likelihood of positive selection acting on individual codons in a coding sequence. Our analysis finds a very strong and significant footprint of positive selection acting on the functional HopZ1 alleles (Table 1), with 7.2% of codons having an equilibrium dN/dS = 6.69 (model M3). These patterns are particularly pronounced in the C- terminus of the protein following the *hopZ1c* frameshift insertion (Figure 4). Of the 22 positively selected residues, 17 fall after the frameshift mutation, and eight of these follow the premature stop in *hopZ1c*. An analysis using only the *hopZ1a* and *hopZ1b* alleles found many of the same amino acids to be under selection, indicating that while the 19 amino acids in HopZ1c that follow the frameshift strongly contribute to the signature of selection, they are by no means solely responsible for it. Of the five codons in the N-terminal and central portion of the protein identified as being under positive selection, four of these are located very close (between three and nine amino acids) to one of the amino acids in the cysteine–protease catalytic triad, suggesting that these amino acids may be involved in protein–substrate interaction.

**Table 1:**
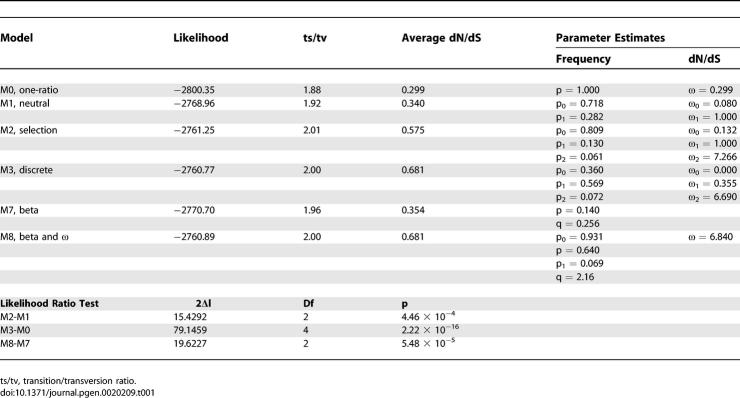
PAML Selection Analysis

### HopZ Alleles Are Cysteine Proteases

**Figure 5:**
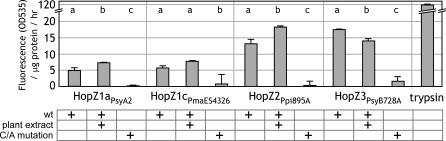
Protease Activity of HopZ T3SEs in P. syringae Protease activity of HopZ1a_PsyA2_, HopZ1c_PmaES4326_, HopZ2_Ppi895A_, and HopZ3_PsyB728a_ measured using Invitrogen RediPlate 96 EnzChek Protease Assay Kit green fluorescence is shown as fluorescence production (OD 535) after a 1-h incubation of the purified His-tag fusion proteins with a green fluorescence–labeled substrate at room temperature. Trypsin was used as a positive control. The grid along the bottom of the figure indicates if the wild-type protein was used, if plant extract was added to the reaction, or if the cysteine-to-alanine mutant protein was used. The error bars indicate standard errors between two replicates. Each experiment was repeated three times with similar results except for HopZ3_PsyB728a_, which gave inconsistent results due to instability of the protein. A gelatine in-gel protease assay was performed to confirm the enzymatic activity of HopZ3_PsyB728a_. Letters above the histogram bars indicate significance classes from a Fisher's Least Significant Difference test. wt, wild-type.

While it has been shown that the *Yersinia* YopJ T3SEs are cysteine proteases [12] or acetyltransferases [15], this has not yet been shown for their P. syringae HopZ homologs. Furthermore, the intense selective pressure experienced by HopZ1, which has resulted in a dramatic increase in the rate of nonsynonymous substitutions at the C terminus of HopZ1a and HopZ1b, and perhaps in a frameshift in HopZ1c, raises the possibility that the HopZ alleles may have functionally diverged. To test this, we purified recombinant *P. syringae* HopZ T3SEs and assayed their activity using a fluorescence-based protease assay (Figure 5). The enzymatic activities of HopZ1a_PsyA2_ and HopZ1c_PmaES4326_ are similar, while that of HopZ2_Ppi895A_ is approximately 2-fold higher. The addition of N. benthamiana leaf tissue extract to the assays increased the activity of all three enzymes, suggesting that plant cofactors may be required for full cysteine–protease activity. In contrast to HopZ1 and HopZ2, the protease activity of HopZ3_PsyB728a_ could not be consistently measured with this assay; therefore, a gelatine in-gel protease assay was used to confirm its activity. The wild-type HopZ3_PsyB728a_ showed strong band clearing, indicating protease activity, although no increase in activity was detected when plant extract was added (unpublished data). No protease activity was detected via the gelatine assay for HopZ1a_PsyA2_, HopZ1c_PmaES4326_, and HopZ2_Ppi895A_. We confirmed the role of the conserved cysteine residue that makes up part of the cysteine protease catalytic triad by testing the activity of mutated HopZ family T3SEs in which the conserved cysteine was changed to alanine. Mutation of the conserved cysteine residue completely abolished protease activity in all assays (Figure 5). These results confirm that all of the HopZ family members are cysteine proteases, although the substrate specificity of HopZ3 may differ.

### HopZ Alleles Elicit Defense Responses in a Host-Specific Manner

**Figure 6:**
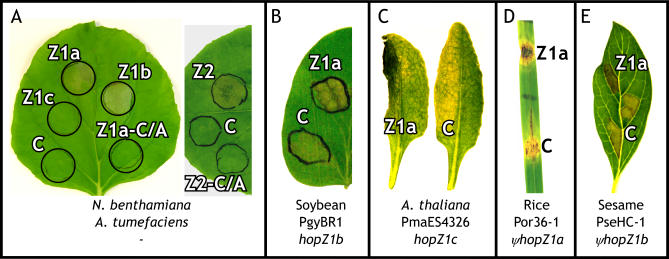
In Vivo Defense Induction Assays The plant host, infiltrated strain, and *hopZ* allele carried by the infiltrated strain are indicated below each leaf image. (A–E) Z1a, Z1b, Z1c, and Z2 represent the infiltration zones of HopZ1a, HopZ1b, HopZ1c, and HopZ2, respectively. Z1a-C/A and Z2-C/A identify the infiltration zone of the HopZ1a and HopZ2 alleles carrying the cysteine-to-alanine replacement in the catalytic core, respectively. C represents the vector control. In all panels HopZ1a induces an HR, while the other infiltrations either result in disease symptoms or no response. (A) Transient expression assay using N. benthamiana leaves infiltrated with A. tumefaciens C58C1(pCH32), carrying HopZ1 or HopZ2 T3SEs in pMDD1 vector. (B) Soybean (Glycine max cv. OAC Bayfield) leaves infiltrated with PgyBR1, carrying pUCP20 or pUCP20HopZ1a*_ PsyA2_*. PgyBR1 carries *hopZ1b* natively. (C) A. thaliana (column 1) leaves infiltrated with PmaES4326, carrying pUCP20 or pUCP20HopZ1a*_ PsyA2_*. PmaES4326 carries *hopZ1c* natively. (D) Rice (O. sativa cv. Jefferson) leaves infiltrated with Por36–1 carrying pUCP20 or pUCP20HopZ1a*_ PsyA2_*. Por36–1 carries ψ*hopZ1a* natively. (E) Sesame (S. indicum) leaves infiltrated with PseHC-1 carrying pUCP20 or pUCP20HopZ1a*_ PsyA2_*. PseHC-1 carries ψ *hopZ1b* natively.

How does the genetic variation observed among the HopZ alleles affect the ability of strains to attack their respective host plants? To determine how the ancestral *hopZ1a* allele influences the interactions between strains carrying derived HopZ alleles and their respective hosts, we transformed P. syringae isolates PgyBR1 and PmaES4326, which endogenously carry *hopZ1b* and *hopZ1c,* respectively, with the full-length *hopZ1a*_PsyA2_ under the control of its native promoter. These transgenic strains were then infiltrated into their respective plant hosts (soybean and A. thaliana), and the responses of the plants were monitored. Both transformed bacteria elicited a hypersensitive response (HR)—a visible and characteristic component of the plant defense response (Figure 6B and 6C).

To test if the ancestral *hopZ1a* allele is detrimental in the strains carrying degenerate *hopZ1* alleles, we transformed PseHC-1 (a sesame pathogen carrying ψ*hopZ1b)* and Por36–1 (a rice pathogen carrying ψ*hopZ1a*) with *hopZ1a_PsyA2_* and infiltrated them into their respective hosts. Whereas the wild-type strains cause disease symptoms in their hosts, both transformed strains induce an HR (Figure 6D and 6E). These data indicate that an R protein–mediated defense response is induced by the ancestral HopZ1a, but not for all of the derived homologs, even though all of the T3SEs retain cysteine–protease activity. The ability of plant hosts to recognize and respond to the different HopZ alleles is strongly host-dependent.

We used *Agrobacterium*-mediated transient expression to further examine the interaction between bacterial and host proteins. This technique permits the expression of individual genes directly inside the host cell, thereby eliminating other variables introduced by the pathogen–host interaction. Transient expression of HopZ1a, HopZ1b, and HopZ2 induced an HR in *N. benthamiana,* while HopZ1c did not (Figure 6A). HopZ3 also did not induce an HR in N. benthamiana when expressed either with or without its chaperone (unpublished data), which is consistent with the findings of Deng et al. [20]. These data indicate that specific HopZ alleles induce differential responses in a host-dependent manner, and may therefore contribute to host specificity.

The positively selected genetic variation observed at the C-terminus of HopZ1 may be important either for avoiding recognition by host-resistance proteins or for virulence-target specificity. While it is possible that the as-yet-unidentified R protein directly recognizes the C-terminus of the HopZ1a protein, *Agrobacterium*-mediated transient assays expressing a mutated *hopZ1a* allele with a cysteine-to-alanine replacement in the catalytic triad did not induce an HR in N. benthamiana (Figure 6A). The same results were obtained with HopZ2 and the HopZ2 cysteine-to-alanine catalytic mutation (Figure 6A). This confirms that the cysteine–protease function is required for the R protein–mediated defense induction. These data are consistent with the guard hypothesis of R-protein action [45], which predicts that it is the virulence function of the bacterial effector that induces the defense response. Since it is the proteolytic function that is recognized by the R protein, the high C-terminal diversity may instead be important for virulence-target specificity.

### Evolutionary Implications

**Figure 7:**
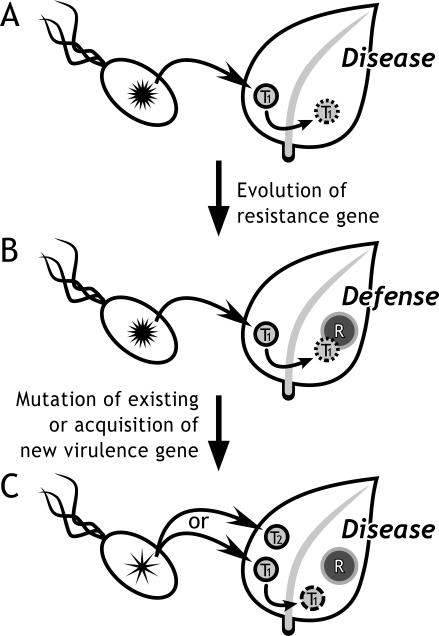
Arms-Race Model (A–C) Star-shaped figures in bacteria represent T3SEs. T_1_ and T_2_ are two virulence targets in the host. R is a resistance protein that induces the host-defense response upon recognition of a plant-virulence target modified by a bacterial effector. (A) The pathogen secretes the ancestral HopZ1a T3SE which modifies a host-virulence target and contributes to the disease process. (B) The plant host evolves or acquires an R protein that recognizes the modified target of HopZ1a, and uses this as a cue to induce the defense response, making the plant host resistant to the pathogen. (C) Strong positive selection imposed by the host R protein results in the evolution of modified HopZ T3SEs (e.g., HopZ1b and HopZ1c) or the acquisition of homologs from ecologically similar plant pathogens (e.g., HopZ2 and HopZ3). These T3SEs retain cysteine-protease function, but are not recognized by the R protein, and therefore do not induce a defense response. They may avoid R-protein recognition by either attacking a new virulence target in the host, or by modifying the original target so that it is not recognized by the R protein.

A coevolutionary arms race requires that there be reciprocal selective pressures imposed on two or more species, leading to escalatory adaptations. Escalatory adaptations are those in which evolutionary changes in the pathogen are matched by evolutionary changes in the host, such that the relative balance between these organisms is maintained. In general, these criteria are difficult to meet since they require information about past adaptations and interactions. By combining evolutionary and functional approaches, we have been able to identify a coevolutionary arms race and describe a mechanism for its action (Figure 7). Phylogenetic and population genetic analyses indicate that *hopZ1a* most closely resembles the ancestral *P. syringae hopZ* cysteine protease. The ancient bacterium that expressed this T3SE must have attacked a host that lacked a cognate R protein for HopZ1a. Over time, either this ancestral host evolved or acquired the appropriate R protein, or the ancestral pathogen began infecting host species that already had the appropriate R protein. This R protein imposed strong selective pressures on HopZ1a, which resulted in either gene loss, extensive mutational change (pathoadaptation), or the recruitment of homologs from other ecologically similar species by horizontal gene transfer. These new T3SEs retain their cysteine–protease function, but are not all recognized by the R protein that responds to the ancestral HopZ1a. While none of the derived alleles induce a defense response in their respective hosts, the ancestral allele does, indicating that this arms race has selected for alleles that can avoid or suppress host recognition. It is important to note that these evolutionary dynamics likely occurred over many millions of years, and without information regarding the host targets and resistance genes it is impossible to determine the relative importance of host coevolution versus host switching. An important area of future research will be to determine if these T3SEs modify different virulence targets in their hosts, as suggested by the HopZ3 substrate specificity results, or, alternatively, whether the original virulence target is modified in such a way that it does not induce R-protein signaling. Furthermore, it will be very interesting to identify the resistance protein that recognizes these T3SEs, and to perform parallel evolutionary analyses.

In this study we find a diversity of functional HopZ homologs that are able to evade the detection of R proteins, as well as degenerate alleles, suggesting that altering the effector rather than losing it may be an important strategy utilized by bacterial pathogens to overcome host recognition. It is particularly noteworthy that these alternative allelic forms have evolved both through pathoadaptive change via the mutational process, as well as through horizontal transfer. Horizontal gene transfer and gene loss are well-known and accepted mechanisms for modifying T3SE-mediated host-specific interactions [11,27,28,33,46], yet pathoadaptation in this context is less well-understood. While pathoadaptation is well-established in other virulence-associated systems such as the *Escherichia coli fimH* loci [29,30], it has not yet been shown to influence T3SE–host interactions. The closest examples to pathoadaptive T3SE mutations are the replication-slippage mutations of the 102-bp repeat in the *avrBs3* locus from X. campestris [34], and the insertion of mobile elements into Ralstonia solanacearum and X. campestris T3SEs [36–38]. Perhaps more telling is the work of Stevens et al. [39], who identified multiple alleles of the P. syringae T3SE, AvrPphE, which are recognized by an R protein carried by different bean cultivars. Unfortunately, the importance of this natural variation is difficult to ascertain since the T3SEs were expressed from strong constitutive promoters in all of the interaction studies.

There is substantial debate over the relative importance of the population genetics arms-race model verses the trench-warfare model in describing pathogen–host interactions [7,27,42,47–51]. These discussions specifically frame the arms-race model as a continual selective turnover of alleles driven by selective sweeps of favored alleles, and the trench-warfare model as a dynamic maintenance of genetic variation driven by changes in resistance-allele frequencies [7]. The former scenario emphasizes positive selection, while the latter predicts some form of balancing or diversifying selection. While the strong positive selection seen in *hopZ1* appears to support the arms-race model, the extensive genetic variation evident in the HopZ family as a whole could be seen as supporting the trench warfare model. As is often the case, both models likely describe some aspect of these interactions. The particular model that is dominant may depend upon the scale of examination.

This study sheds new light on the role of natural genetic variation in pathogen–host interactions, and provides insight into both the evolutionary and functional mechanisms underlying coevolutionary interactions. It provides a clearer picture of the molecular interactions that drive arms races, and insight into how ecological processes play out at the molecular and evolutionary scale.

## Materials and Methods

### Bacterial strains.

P. syringae isolates (Table S1) were grown in King's B medium at 30 °C. Minimal medium supplemented with fructose was used to induce the expression of the T3SEs [52].

### HopZ characterization.

Genes encoding the T3SEs *hopZ1a, hopZ1c, hopZ2,* and *hopZ3* were obtained by PCR (primers available from authors upon request). Genomic DNA from 96 P. syringae strains was prepared using the PureGene DNA isolation protocol (Gentra Systems, http://www.gentra.com). according to the manufacturer's instructions. The DNA was digested and hybridized with the four *hopZ* genes as probes (Gene Images CDP-*Star*, Amersham Biosciences, http://www4.amershambiosciences.com). *hopZ* genes were isolated out of all of positive strains by PCR, inverse PCR, and/or molecular cloning to obtain the full-length sequence and the flanking regions. *hopZ3* is in an operon immediately downstream of its chaperone. All the *hopZ3* alleles were cloned with their chaperones. Multiple clones and PCR products were sequenced for each allele following manufacturer's protocols on a Beckman Coulter CEQ8000 DNA analyzer (Beckman Coulter, http://www.beckmancoulter.com). The promoters were examined for a *hrp* promoter box, the conserved sequence that directs the transcription of P. syringae T3SEs [53]. The expression of all T3SEs was confirmed under T3SS-inducing conditions by RT-PCR and Northern blot analysis (Figure S1). PCR products and clones were sequenced following manufactures protocols on a Beckman-Coulter CEQ8000 DNA analyzer. GenBank accession numbers for all of the sequenced alleles are presented in Table S2.

### Protein purification and protease-activity assay.

*hopZ* genes (*hopZ3* was cloned without its chaperone) and their cysteine-to-alanine replacement mutants were amplified by PCR and cloned into the pET14b vector (Novagen, http://www.emdbiosciences.com). The His-tagged fusion proteins were overexpressed in E. coli BL21 cells (Novagen), purified using nickel columns, and dialyzed in reaction buffer (100 mM Tris [pH 8.0], 150 mM NaCl, 1 mM DTT). Protease activity was measured using both RediPlate 96 EnzChek Protease Assay Kit green fluorescence (Invitrogen, http://invitrogen.com) and a gelatine in-gel assay. Plant extract was obtained from N. benthamiana by grinding leaf tissue in the reaction buffer and then spinning at 14,000 rpm for 10 min. 100 ng of plant extract was added to each reaction. The gelatine in-gel assays were performed as previously described [54]. Type A gelatine from porcine skin (Sigma-Aldrich Inc., http://www.sigmaaldrich.com) was added to 10% acrylamide to a final concentration of 1 mg/mL to make gelatine-polyacrylamide gels. Native protein samples (with and without plant extract) were loaded on the gel, which was run as a regular SDS-PAGE. After running, the gel was incubated at 30 °C in 2.5% Triton X-100 for 2 h, and then switched to a protease-activation buffer (50 mM sodium phosphate buffer [pH6.8], 0.1% Triton X-100, 5 mM L-cysteine) overnight. Degradation of gelatine was detected by staining the gel in Coomassie Blue.

### *Agrobacterium*-mediated transient-expression assay.

*hopZ* genes were cloned into pMDD1 vector [55], and transformed into Agrobacterium tumefaciens C58C1 (pCH32). *hopZ3* was cloned both with and without its upstream chaperone for these assays. The resulting bacteria were pressure-infiltrated into 3-wk-old N. benthamiana leaves [14]. Plants were kept at room temperature under continuous low light for 48 h before HR was scored.

### Hypersensitive response assays.

*hopZ1a_PsyA2_* with its native promoter was cloned into broad–host range vector pUCP20 to construct pUCP20HopZ1a*_PsyA2_*. This plasmid was then transformed into P. syringae isolates PgyBR1, PmaES4326, PseHC-1, and Por36–1 by electroporation (2.5 kV, 25 μF, 600 Ω). The resulting bacteria were hand-infiltrated into leaves of soybean, *A. thaliana*, sesame, and rice respectively. Soybean (Glycine max cv. OAC Bayfield) was grown in a growth chamber at 22 °C with a light regime of 16/8 h for 21 d. The unifoliars were infiltrated with a 5 × 10^8^ cfu/mL suspension of bacteria, and HR was scored after 48 h. Sesame (Sesamum indicum) was grown in the greenhouse for 6 wk before the primary leaves were infiltrated with a 1 × 10^7^ cfu/mL suspension of bacteria. The HR was scored after 24 h. Rice plants (Oryza sativa cv. Jefferson) were grown in a growth chamber at 26–28 °C at 80% humidity for 7 d, and the first leaf of each plant was infiltrated with a 5 × 10^7^ cfu/mL bacteria suspension. The infiltrated plants were kept at room temperature with continuous light for 24 h, and then transferred back to the growth chamber for an additional 2–48 h before the HR was scored. The HR assay on A. thaliana eco. Col was performed as previously described [56]. All assays were performed in triplicate along with pUCP20 [57] as controls.

### Data analysis.

DNA sequence data was manually analyzed and edited with BioEdit version 7.0 (http://www.mbio.ncsu.edu/BioEdit/bioedit.html). Alignments were made in ClustalX version 1.83 [58] and Dialign2 [59], and manipulated in GeneDoc version 2.6 (www.psc.edu/biomed/genedoc). Phylogenetic analyses were performed in MEGA version 3.1 [60] and Phylip version 3.65 [61]. All trees were constructed using both neighbor-joining and maximum-likelihood methods. Nucleotide trees were constructed with the Kimura two-parameter model using a 2:1 transition–transversion rate and gamma correction for rate heterogeneity (α = 0.2). Protein trees were constructed with the JTT substitution matrix. Bootstrapping was performed with 1,000 pseudoreplicates. All neighbor-joining and maximum-likelihood trees were entirely congruent with respect to their major branching; therefore, only neighbor-joining trees are presented. Population-genetic analyses were performed with DnaSP [62] and MEGA. Split decomposition network analysis was performed via SplitsTree version 3.2 [63,64].

PAML version 3.15 [44] analyses were performed using the codeml module. Models M0 (one-ratio), M1 (neutral), M2 (selection), M3 (discrete), M7 (beta), and M8 (beta + ω) were analyzed. PHYIP was used to generate a maximum-likelihood tree for the treefile. The following parameters settings were used in the control file: CodonFreq = 2, clock = 0, aaDist = 0, model = 0, cleandata = 0, fix_kappa = 0, fix_omega = 0. Likelihood ratio tests were performed to determine the relative significance of the models using the degrees of freedom specified in Table S2.

## Supporting Information

Figure S1 Expression Analysis of HopZ T3SEs (A) RT-PCR analysis. (B) Northern analysis. u, uninduced; i, induced for T3SS expression.
(3.6 MB TIF)

Figure S2 Gene Genealogy of the HopZ Family Midpoint-rooted neighbor-joining phylogenetic analysis of all HopZ T3SEs in *P. syringae,* based on the DNA sequence. Numbers above nodes represent 1,000 bootstrap replicates. Only those values with >60% support are shown.
(2.1 MB TIF)

Figure S3 Protein Multiple Sequence Alignment of All HopZ1 Alleles Shading indicates the degree of conservation. The arrows indicate the three amino acids (H, histidine; E, glutamic acid; C, cysteine) that form the catalytic triad.
(3.6 MB TIF)

Figure S4 Split Decomposition Network Phylogenetic Analysis of the *hopZ1* Homologs Numbers along the branches are bootstrap scores from 1,000 pseudoreplicates. Only those bootstrap scores above 50 are shown.
(1.3 MB TIF)

Table S1 Pseudomonas syringae Strains (237 KB DOC)

Table S2 GenBank Accession Numbers (58 KB DOC)

## Abbreviations

bp: base pair
HR: hypersensitive response
MLST: multilocus sequence typing
T3SE: type III secreted effector
T3SS: type III secretion system
