# Supplementary material for: Type III Effector Diversification via Both Pathoadaptation and Horizontal Transfer in Response to a Coevolutionary Arms Race
Source: PLoS Genet. 2006 Dec 22;2(12):e209. doi: 10.1371/journal.pgen.0020209 (PMC1713259; doi:10.1371/journal.pgen.0020209)
Supplement: Supplementary file 1 [file pgen.0020209.st001.doc]

| Supplemental Table 1. *Pseudomonas syringae* strains | | | | | | | | |
| --- | --- | --- | --- | --- | --- | --- | --- | --- |
| Pathovar | Designation | Strain Name | | Place Isolation | Year Isolation | Host | Source^1^ | Accession^1^ |
| aceris | ATCC10853 | Pac | A10853 |  |  | maple | J.Dangl | ATCC10853 |
| actinidiae | FTRS_L1 | Pan | FTRS_L1 | Japan | 1984 | kiwi | MAFF | MAFF302091 |
| aesculi | 0893_23 | Pae | 0893_23 | USA |  | horse chestnut | D.Cooksey |  |
| apii | 1089_5 | Pap | 1089_5 | USA |  | celery | D.Cooksey |  |
| aptata | 601 | Ptt | 601 |  | 1966 | sugarbeet | MAFF | MAFF301008 |
| aptata | DSM50252 | Ptt | DSM5022 |  |  | wheat | J.Dangl |  |
| aptata | G733 | Ptt | G733 |  | 1976 | brown rice | MAFF | MAFF302831 |
| atrofaciens | DSM50255 | Paf | DSM5025 |  |  | wheat | J.Dangl |  |
| broussonetiae | KOZ8101 | Pbr | KOZ8101 | Japan | 1980 | paper mulberry | MAFF | MAFF810036 |
| cilantro | 0788_9 | Pci | 0788_9 | USA |  | cilantro | D.Cooksey |  |
| coronafaciens | 3113 | Pcn | 3113 | UK | 1958 | oats | D. Arnold | ICMP3113 |
| coronafaciens | KN221 | Pcn | KN221 |  | 1984 | oats | MAFF | MAFF302787 |
| glycinea | BR1 | Pgy | BR1 |  | 1989 | soybean | MAFF | MAFF210373 |
| glycinea | KN127 | Pgy | KN127 |  | 1982 | soybean | MAFF | MAFF302751 |
| glycinea | KN166 | Pgy | KN166 |  | 1982 | soybean | MAFF | MAFF302770 |
| glycinea | KN28 | Pgy | KN28 |  | 1981 | soybean | MAFF | MAFF302676 |
| glycinea | KN44 | Pgy | KN44 | Japan | 1981 | soybean | MAFF | MAFF301683 |
| glycinea | LN10 | Pgy | LN10 |  | 1989 | soybean | MAFF | MAFF210389 |
| glycinea | MAFF301765 | Pgy | M301765 |  | 1982 | soybean | MAFF | MAFF301765 |
| glycinea | MOC601 | Pgy | MOC601 |  | 1994 | soybean | MAFF | MAFF311113 |
| glycinea | R4a | Pgy | R4a |  |  | soybean | J.Dangl |  |
| glycinea | UnB647 | Pgy | UnB647 |  |  | kidney bean | MAFF | MAFF210405 |
| japonica | MAFF301072 | Pja | M301072 | Japan | 1951 | barley | MAFF | MAFF301072 |
| lachrymans | 107 | Pla | 107 |  |  | cucumber | J.Dangl | MAFF301315 |
| lachrymans | 3988 | Pla | 3988 | USA | 1935 | cucumber | D. Arnold | ICMP3988 |
| lachrymans | 1188_1 | Pla | 1188_1 | USA |  | zucchini | D.Cooksey |  |
| lachrymans | ATCC7386 | Pla | A7386 |  |  | cucumber | J.Dangl | MAFF302278 |
| lachrymans | N7512 | Pla | N7512 | Japan | 1975 | cucumber | MAFF | MAFF301315 |
| lachrymans | YM7902 | Pla | YM7902 |  | 1979 | cucumber | MAFF | MAFF730057 |
| lachrymans | YM8003 | Pla | YM8003 |  | 1980 | cucumber | MAFF | MAFF730069 |
| maculicola | 4981 | Pma | 4981 | Zimbabwe |  | cauliflower | D.Cuppels |  |
| maculicola | AZ85297 | Pma | AZ85297 |  | 1985 | Chinese cabbage | MAFF | MAFF302539 |
| maculicola | ES4326 | Pma | ES4326 |  | 1965 | radish | J.T.Greenberg |  |
| maculicola | H7311 | Pma | H7311 | Japan | 1973 | Chinese cabbage | MAFF | MAFF301174 |
| maculicola | H7608 | Pma | H7608 |  | 1976 | Chinese cabbage | MAFF | MAFF301175 |
| maculicola | KN203 | Pma | KN203 |  | 1983 | Chinese cabbage | MAFF | MAFF302783 |
| maculicola | KN84 | Pma | KN84 |  | 1982 | radish | MAFF | MAFF302724 |
| maculicola | KN91 | Pma | KN91 |  | 1982 | radish | MAFF | MAFF302731 |
| maculicola | M4a | Pma | M4a |  |  | radish | J.Dangl |  |
| maculicola | M6 | Pma | M6 | UK | 1965 | cauliflower | J.Dangl |  |
| maculicola | YM7930 | Pma | YM7930 |  | 1979 | radish | MAFF | MAFF301419 |
| mellea | N6801 | Pme | N6801 |  | 1968 | tobacco | MAFF | MAFF302303 |
| mori | MAFF301020 | Pmo | M301020 | Japan | 1966 | mulberry | MAFF | MAFF301020 |
| morsprunorum | 19322 | Pmp | 19322 |  |  | European plum | J.Dangl | ATCC19322 |
| morsprunorum | FTRS_U7805 | Pmp | FTRS_U7 | Japan | 1978 | Japanese apricot | MAFF | MAFF301436 |
| myricae | AZ84488 | Pmy | AZ84488 |  | 1984 | bayberry | MAFF | MAFF302460 |
| myricae | MAFF302941 | Pmy | M302941 |  | 1989 | bayberry | MAFF | MAFF302941 |
| oryzae | 36_1 | Por | 36_1 |  | 1983 | rice | MAFF | MAFF301538 |
| oryzae | I_6 | Por | I_6 |  | 1991 | rice | MAFF | MAFF311107 |
| phaseolicola | 1302A | Pph | 1302A | Ethiopia | 1984 | kidney bean | D.Arnold |  |
| phaseolicola | 1449B | Pph | 1449B | Ethiopia | 1985 | hyacinth bean | D.Arnold |  |
| phaseolicola | HB10Y | Pph | HB10Y |  |  | snap bean | P. Turner | ATCC21781 |
| phaseolicola | KN86 | Pph | KN86 | Japan | 1982 | kidney bean | MAFF | MAFF301673 |
| phaseolicola | NPS3121 | Pph | NPS3121 |  |  | kidney bean | J.T.Greenberg |  |
| phaseolicola | NS368 | Pph | NS368 |  | 1992 | kidney bean | MAFF | MAFF311004 |
| phaseolicola | R6a | Pph | R6a |  |  | kidney bean | J.Dangl |  |
| phaseolicola | SG44 | Pph | SG44 | USA | 1980 | snap bean | S.Hirano |  |
| phaseolicola | Y5_2 | Pph | Y5_2 |  |  | kudzu | MAFF | MAFF311162 |
| pisi | 895A | Ppi | 895A |  |  | pea | D. Arnold |  |
| pisi | H5E1 | Ppi | H5E1 |  | 1993 | pea | MAFF | MAFF311141 |
| pisi | H5E3 | Ppi | H5E3 |  | 1993 | pea | MAFF | MAFF311143 |
| pisi | H6E5 | Ppi | H6E5 |  | 1994 | pea | MAFF | MAFF311144 |
| pisi | H7E7 | Ppi | H7E7 |  | 1995 | pea | MAFF | MAFF311146 |
| pisi | PP1 | Ppi | PP1 | Japan | 1978 | pea | MAFF | MAFF301208 |
| pisi | R6a | Ppi | R6a |  |  | pea | J.Dangl |  |
| savastanoi | 4352 | Psv | 4352 | Yugoslavia |  | olive | A. Colmer |  |
| sesame | HC_1 | Pse | HC_1 |  |  | sesame | MAFF | MAFF311181 |
| syringae | 1212R | Psy | 1212R |  |  | pea | D.Arnold |  |
| syringae | A2 | Psy | A2 |  |  | ornamental pear | C.Bender |  |
| syringae | B48 | Psy | B48 | USA |  | peach | T. Denny |  |
| syringae | B64 | Psy | B64 | USA |  | wheat | T. Denny |  |
| syringae | B728A | Psy | B728A | USA |  | snap bean | S.Hirano |  |
| syringae | B76 | Psy | B76 | USA |  | tomato | T. Denny |  |
| syringae | FF5 | Psy | FF5 | USA | 1998 | ornamental pear | C.Bender |  |
| syringae | FTRS_W6601 | Psy | FTRS_W6 |  | 1966 | Japanese apricot | MAFF | MAFF301429 |
| syringae | FTRS_W7835 | Psy | FTRS_W7 |  | 1978 | Japanese apricot | MAFF | MAFF301430 |
| syringae | L177 | Psy | L177 |  | 1983 | lilac | MAFF | MAFF302085 |
| syringae | LOB2_1 | Psy | LOB2_1 | Japan | 1986 | lilac | MAFF | MAFF301861 |
| syringae | NCPPB281 | Psy | NCPPB28 | UK |  | lilac | B.Hancock | ATCC19310 |
| syringae | Ps9220 | Psy | Ps9220 |  | 1992 | spring onion | MAFF | MAFF730125 |
| syringae | PSC1B | Psy | PSC1B | USA |  | corn | T. Denny |  |
| tabaci | 6606 | Pta | 6606 | Japan | 1967 | tobacco | MAFF | MAFF301612 |
| thea | K93001 | Pth | K93001 |  | 1993 | tea | MAFF | MAFF302851 |
| tomato | 487 | Pto | 487 | Greece |  | tomato | D.Cuppels |  |
| tomato | 1318 | Pto | 1318 | Switzerland |  | tomato | D.Cuppels |  |
| tomato | 2170 | Pto | 2170 |  | 1984 | tomato | MAFF | MAFF301591 |
| tomato | DC3000 | Pto | DC3000 | UK |  | tomato | J.T.Greenberg |  |
| tomato | DC84_1 | Pto | DC84_1 | Canada |  | tomato | D.Cuppels |  |
| tomato | DC89_4H | Pto | DC89_4H | Canada |  | tomato | D.Cuppels |  |
| tomato | DCT6D1 | Pto | DCT6D1 | Canada |  | tomato | D.Cuppels |  |
| tomato | KN10 | Pto | KN10 |  | 1981 | tomato | MAFF | MAFF302665 |
| tomato | PT23 | Pto | PT23 | USA | 1986 | tomato | N.T. Keen |  |
| tomato | TF1 | Pto | TF1 | USA | 1997 | tomato | S.Hirano |  |
| - | Cit7 | Ps | Cit7 |  |  | navel orange | S.Lindow |  |
| - | TLP2 | Ps | TLP2 |  |  | potato | S.Lindow |  |
| ^1^ MAFF, Japanese ministry of Agriculture, Forestry, and Fisheries. ATCC, American Type Culture Collection. ICMP, International Collection of Micro-organisms from Plants, New Zealand. | | | | | | | | |
