# Supplementary material for: Type III Effector Diversification via Both Pathoadaptation and Horizontal Transfer in Response to a Coevolutionary Arms Race
Source: PLoS Genet. 2006 Dec 22;2(12):e209. doi: 10.1371/journal.pgen.0020209 (PMC1713259; doi:10.1371/journal.pgen.0020209)
Supplement: Supplementary file 2 [file pgen.0020209.st002.doc]

| Supplemental Table 2. GenBank accession numbers | | |
| --- | --- | --- |
| T3SE | Strain | GenBank Accessions |
| hopZ1a | Psy1212R | DQ986440 |
| hopZ1a | PsyPSC1B | DQ986441 |
| hopZ1a | PsyB76 | DQ986442 |
| hopZ1b | PgyBR1 | DQ986431 |
| hopZ1b | PgyUnB647 | DQ986432 |
| hopZ1b | PgyM201765 | DQ986433 |
| hopZ1b | PgyKN44 | DQ986434 |
| hopZ1b | PgyKN127 | DQ986435 |
| hopZ1b | PgyKN166 | DQ986436 |
| hopZ1b | PgyKN28 | DQ986437 |
| hopZ1b | PgyLN10 | DQ986438 |
| hopZ1b | PgyMOC601 | DQ986439 |
| hopZ1c | PmaES4326 | DQ986429 |
| hopZ1c | PmaYM7930 | DQ986430 |
| hopZ1a | PorI_6 | DQ986444 |
| hopZ1a | Por36_1 | DQ986445 |
| hopZ1a | PcnKN221 | DQ986446 |
| hopZ1a | Pcn3113 | DQ986447 |
| hopZ1a | PsyPs9220 | DQ986448 |
| hopZ1b | PseHC_1 | DQ986443 |
|  |  |  |
| hopZ2 | PlaYM8003 | DQ986419 |
| hopZ2 | Pla107 | DQ986420 |
| hopZ2 | PlaYM7902 | DQ986421 |
| hopZ2 | PlaN7512 | DQ986422 |
| hopZ2 | PcoKN221 | DQ986423 |
| hopZ2 | PsyPs9220 | DQ986424 |
| hopZ2 | PseHC_1 | DQ986425 |
| hopZ2 | PpiR6a | DQ986426 |
| hopZ2 | PpiH5E1 | DQ986427 |
| hopZ2 | PacA10853 | DQ986428 |
|  |  |  |
| hopZ3 | PsyFTRSW7835 | DQ986449 |
| hopZ3 | PsyB64 | DQ986450 |
| hopZ3 | PttDSM50252 | DQ986451 |
| hopZ3 | PjaMAFF301072 | DQ986452 |
| hopZ3 | Pla1188_1 | DQ986453 |
| hopZ3 | PmaKN84 | DQ986454 |
| hopZ3 | PmeN6801 | DQ986455 |
| hopZ3 | PanFTRS_L1 | DQ986456 |
